# Supplementary material for: Optimization of single dose VSV-based COVID-19 vaccination in hamsters
Source: bioRxiv. 2021 Sep 3:2021.09.03.458735. Preprint. [Version 1] doi: 10.1101/2021.09.03.458735 (PMC8437312; doi:10.1101/2021.09.03.458735)
Supplement: Supplement 1 — Table S1. Hamster group sizes used in this study. Table S2. Flow cytometry antibodies for hamster samples. Figure S1. Schematic and characterization of VSV-based vaccines. Figure S2. Vaccine-induced cellular immune response in PBMCs. Figure S3. Hamster lung gross pathology after vaccination and challenge with SARS-CoV-2 WA1. Figure S4. Hamster lung gross pathology after vaccination and challenge with SARS-CoV-2 VOC. Figure S5. Histopathology and Immunohistochemistry of hamster lungs with VOC challenge 10 DPV. Figure S6. Schematic presentation of the different immune cells responding to IM and IN vaccination using VSV-based vaccines. [file media-1.pdf]

Optimization of single dose VSV-based COVID-19 vaccination in hamsters

Kyle L. O’Donnell[1], Chad S. Clancy[2], Amanda J. Griffin[1], Kyle Shifflett[1], Tylisha Gourdine[1], Tina Thomas[2], Carrie M. Long[3], Wakako Furuyama[1], and Andrea Marzi[1]*

[1]Laboratory of Virology, [2]Rocky Mountain Veterinary Branch, and [3]Laboratory of Bacteriology, Division of Intramural Research, National Institute of Allergy and Infectious Diseases, National Institutes of Health, Hamilton, MT 59840, USA

Supplemental Materials

Table S1. Hamster group sizes used in this study.
Table S2. Flow cytometry antibodies for hamster samples.
Figure S1. Schematic and characterization of VSV-based vaccines.
Figure S2. Vaccine-induced cellular immune response in PBMCs.
Figure S3. Hamster lung gross pathology after vaccination and challenge with SARS-CoV-2 WA1.
Figure S4. Hamster lung gross pathology after vaccination and challenge with SARS-CoV-2 VOC.
Figure S5. Histopathology and Immunohistochemistry of hamster lungs with VOC challenge 10 DPV.
Figure S6. Schematic presentation of the different immune cells responding to IM and IN vaccination using VSV-based vaccines.

Table S1. Hamster group sizes used in this study.

Group	VSV-SARS2 IN/IM	VSV-SARS2-EBOV IN/IM	Control IN/IM
3 DPV	4/4	4/4	4/4
10 DPV	4/4	4/4	4/4
21 DPV	6/6	8/8	8/8
38 DPV	4/4	4/4	3/3

WA challenge	VSV-SARS2 IN/IM	VSV-SARS2-EBOV IN/IM	Control IN/IM
28 DPV	8/8	8/8	8/8
10 DPV	6/6	6/6	6/6

10 DPV variants	VSV-SARS2 IN	VSV-SARS2-EBOV IN/IM	Control IN/IM
B.1.1.7	6	6/6	3/3
B.1.351	6	6/6	3/3

VSV vesicular stomatitis virus; SARS2 SARS-CoV-2; EBOV Ebola virus; IN intranasal; IM intramuscular; DPV days post-vaccination; WA SARS-CoV-2 Washington isolate (original strain).

Table S2. Flow cytometry antibodies for hamster samples.

Ab target	clone	Ab target	clone	Ab target	clone
CD3	17A2	Ly6C	HK1.4	Ki67	16A8
CD3	KT3.1.1	**Ly6G**	**1A8**	CD19	6D5
Cd49b	Dx5	**CD11b**	**M1/70**	CD27	LG.3A10
**CD69**	**H1.2F3**	CD11c	HL3	CD138	281-2
CD56	809220	**CD4**	**GK1.5**	IL-2	JES6-1A12
Granzyme B	QA16A02	**CD8**	**341**	IL-4	11B11
CD107a	1D4b	CD44	IM7	IA/IE	M5/114.15.2
CD45	30-F11	IFN-γ	XMG1.2	**CD94**	**18d3**
**Live/Dead**		IL-17a	TC11-18H10.1	IA/IE	14-4-4S
**B220**	**RA3-6B2**	CD3	145-2C11		

Ab antibody. All antibodies were tested at dilutions ranging from 1:50 to 1:1,600. Antibodies highlighted in red font were successfully used in our assays.

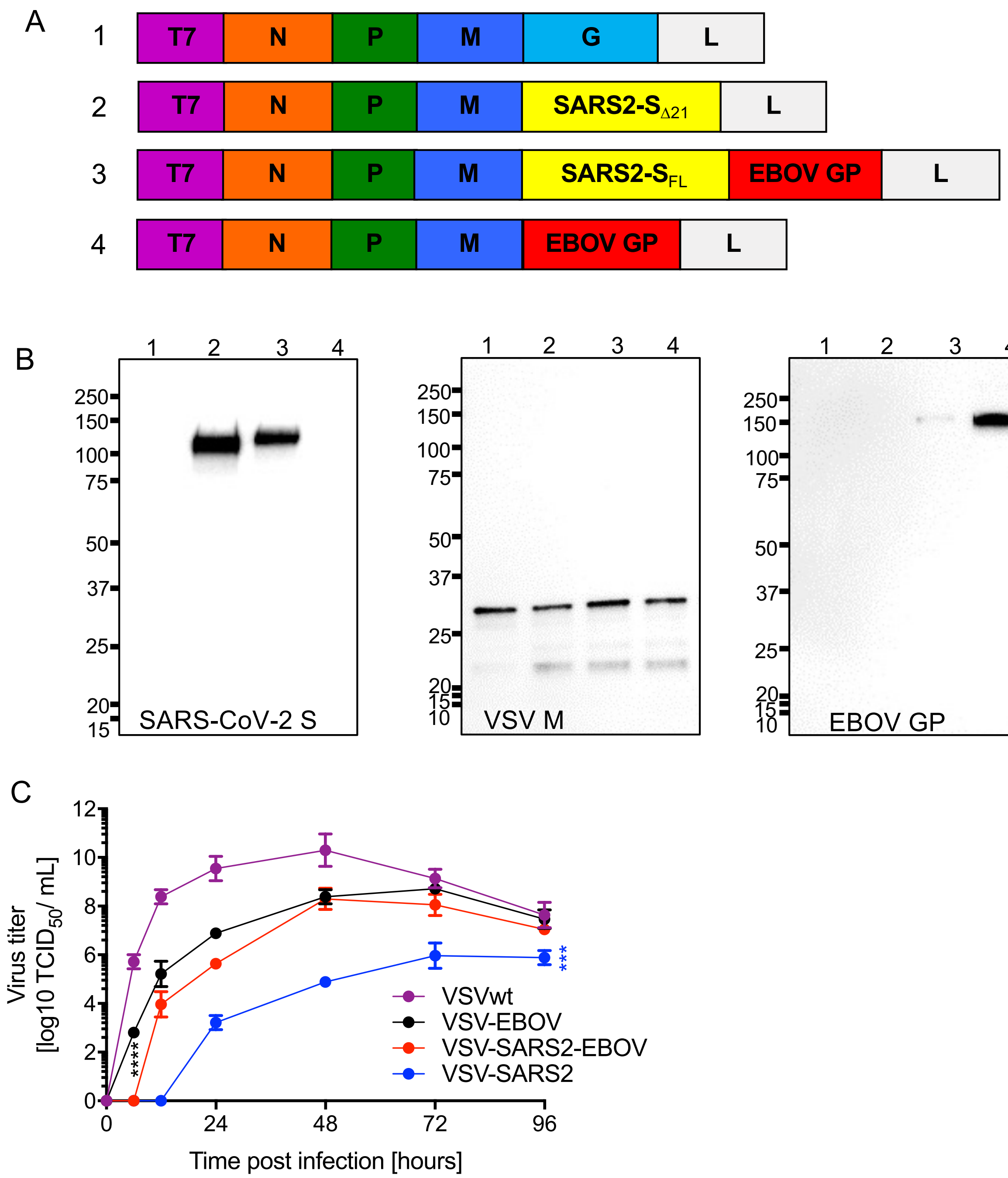

Figure S1. Schematic and characterization of VSV-based vaccines. (A) Schematic illustrating vaccine vector design. T7 promotor; N nucleoprotein; P phosphoprotein; M matrix protein; EBOV GP Ebola virus glycoprotein; L RNA-dependent RNA polymerase; SARS2-S SARS-CoV-2 S. **(B)** Western blot analysis of cell supernatant samples containing VSV vaccines probed for SARS-CoV-2 S (left), VSV M (middle) or EBOV GP (right). 1 VSV wildtype (VSVwt); 2 VSV-SARS2; 3 VSV- SARS2-EBOV; 4 VSV-EBOV. **(C)** Viral growth kinetics on Vero E6 cells. Geometric mean and geometric SD are depicted. Statistical significance as determined by two-way ANOVA with Tukey's multiple comparison is indicated as $p<0.0001$ (****), and $p<0.001$ (***).

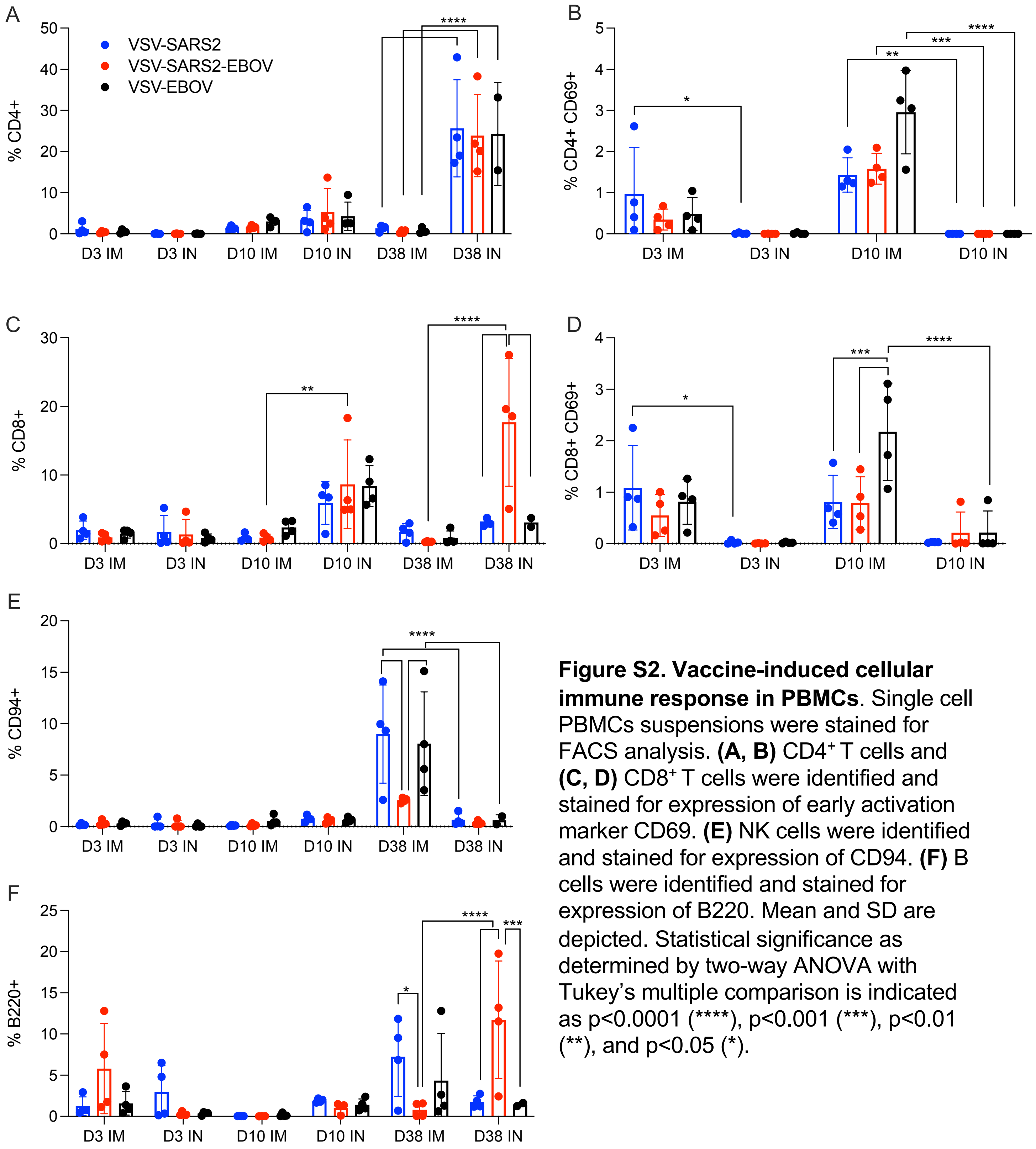

Figure S2. Vaccine-induced cellular immune response in PBMCs. Single cell PBMCs suspensions were stained for FACS analysis. **(A, B)** $CD4^+$ T cells and **(C, D)** $CD8^+$ T cells were identified and stained for expression of early activation marker CD69. **(E)** NK cells were identified and stained for expression of CD94. **(F)** B cells were identified and stained for expression of B220. Mean and SD are depicted. Statistical significance as determined by two-way ANOVA with Tukey's multiple comparison is indicated as p<0.0001 (****), p<0.001 (***), p<0.01 (**), and p<0.05 (*).

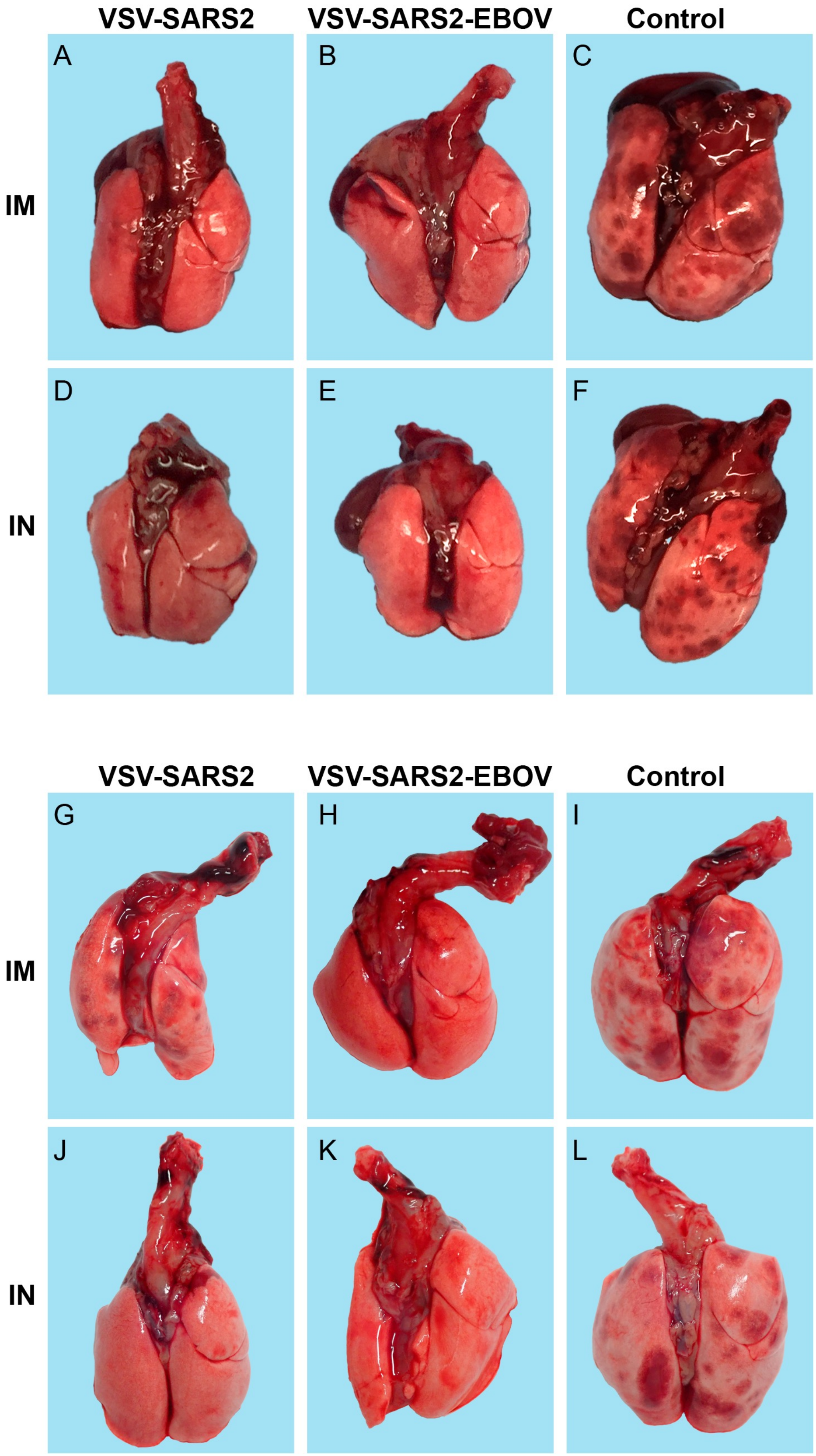

Figure S3. Hamster lung gross pathology after vaccination and challenge with SARS-CoV-2 WA1. Groups of hamsters were vaccinated with a single dose of the indicated vaccine by either the intramuscular (IM) or intranasal (IN) route. Challenge occurred (A-F) 28 days or (G-L) 10 days after vaccination. Representative pictures of hamster lungs with lesions for each vaccine group at 4 days post-challenge.

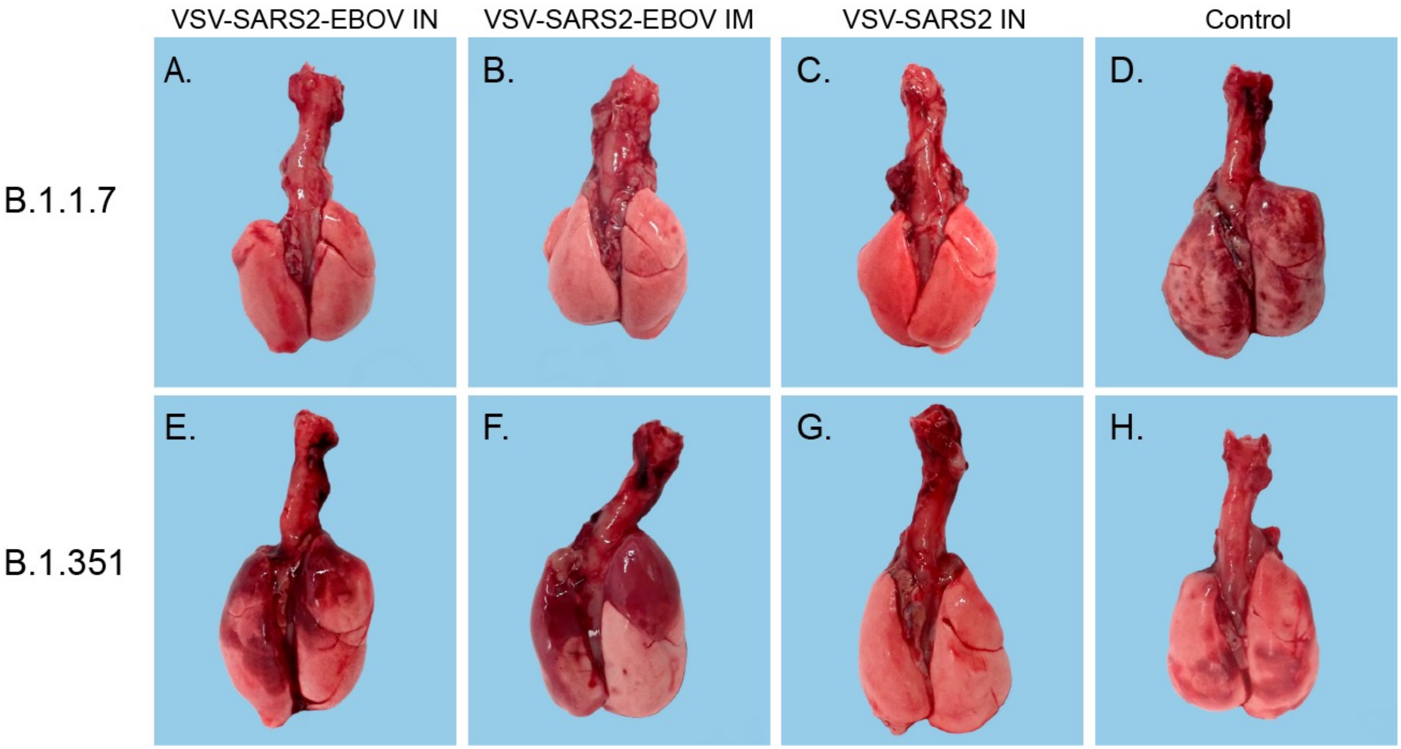

Figure S4. Hamster lung gross pathology after vaccination and challenge with SARS-CoV-2 VOC. Groups of hamsters were vaccinated with a single dose of the indicated vaccine by either the intramuscular (IM) or intranasal (IN) route. Challenge occurred 10 days after vaccination with SARS-CoV-2 B.1.1.7 or B.1.351. Representative pictures of hamster lungs with lesions for each vaccine group at 4 days post-challenge.

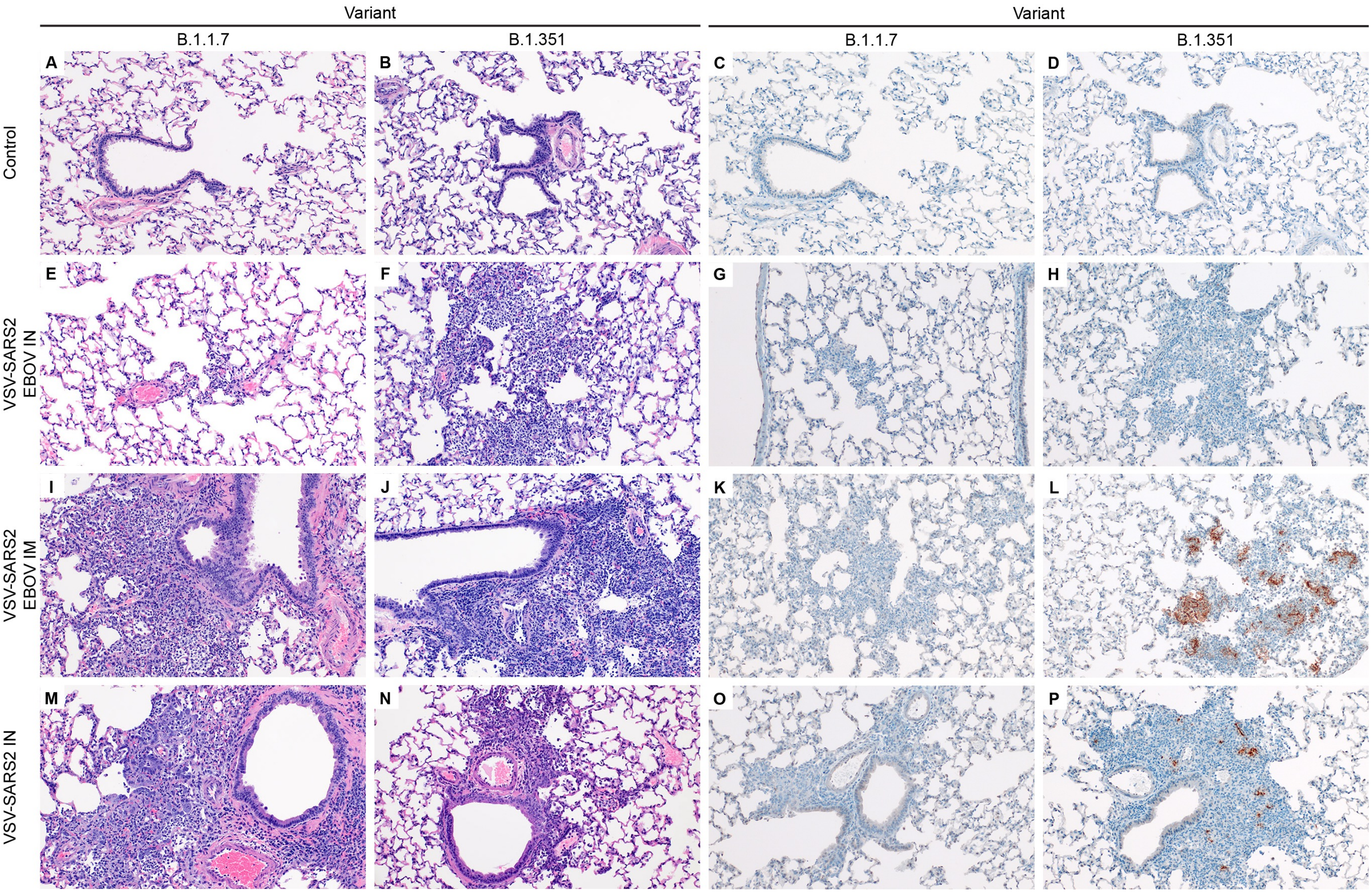

Figure S5. Histopathology and Immunohistochemistry of hamster lungs with VOC challenge 10 DPV. Hamsters were vaccinated 10 days prior to challenge with SARS-CoV-2 B.1.1.7 or B.1.351. At 4 days after challenge lung samples were collected and stained with H&E (A-B,E-F,I-J,M-N) or anti-SARS-CoV-2 nucleocapsid (N) antibody for IHC (C-D,G-H,K-L,O-P). All images are 200x. **(A, B)** Normal pulmonary architecture in a mock-infected hamster. **(C, D)** Normal pulmonary architecture showing lack of SARS-CoV-2 immunoreactivity. **(E)** Rare foci of interstitial pneumonia are observed in VSV-SARS2-EBOV intranasal (IN)-vaccinated hamsters when challenged with the B.1.1.7 variant. **(F)** Rare foci of minimal to mild interstitial pneumonia are observed in VSV-SARS2-EBOV IN-vaccinated hamsters when challenged with the B.1.351 variant. **(G, H)** Lack of SARS-CoV-2 immunoreactivity in foci of minimal to mild interstitial pneumonia in the VSV-SARS2-EBOV IN-vaccinated hamsters following challenge with either B.1.1.7 or B.1.351. **(I, J)** Mild to moderate interstitial pneumonia focused on terminal airways are observed in VSV-SARS2-EBOV intramuscular (IM)-vaccinated hamsters when challenged with B.1.1.7 and B.1.351 variants. **(K)** Lack of SARS-CoV-2 immunoreactivity in foci of interstitial pneumonia in a VSV-SARS2-EBOV IM-vaccinated hamster when challenged with B.1.1.7. **(L)** Abundant SARS-CoV-2 immunoreactivity is observed in type I and II pneumocytes with fewer macrophages in a VSV-SARS2-EBOV IM-vaccinated hamster challenged with B.1.351. **(M, N)** Mild to moderate interstitial pneumonia are observed adjacent to bronchioles and terminal airways in the VSV-SARS2 IN vaccinated hamsters when challenged with either the B.1.1.7 or B.1.351 variants. **(O)** Lack of SARS-CoV-2 immunoreactivity in foci of interstitial pneumonia in a VSV-SARS2 IN-vaccinated hamster when challenged with B.1.1.7. **(P)** Rare SARS-CoV-2 immunoreactivity is observed primarily in type I and II pneumocytes in a VSV-SARS2 IN-vaccinated hamster after B.1.351 challenge.

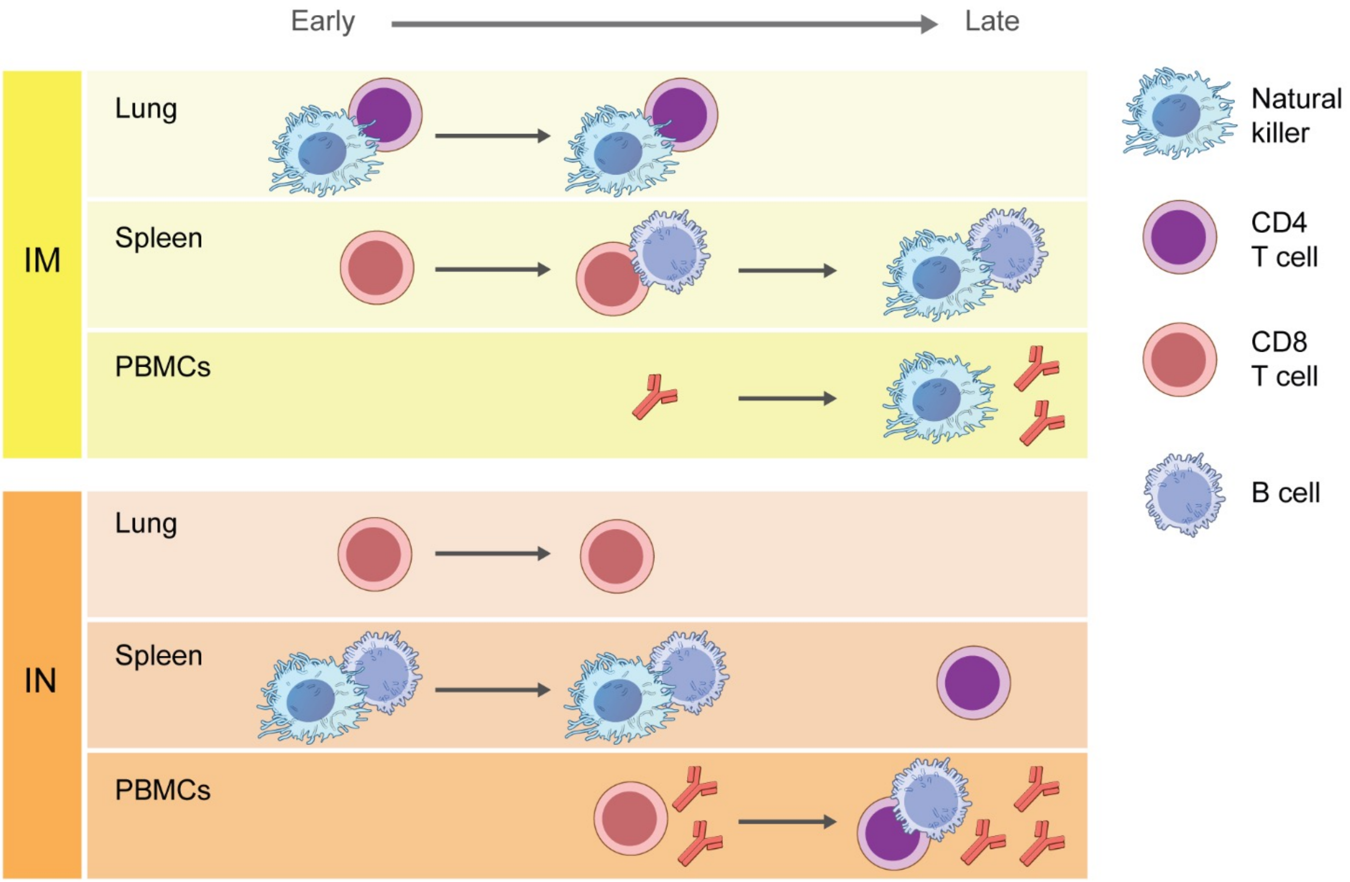

Figure S6. Schematic presentation of the different immune responses to IM and IN vaccination using VSV-based vaccines. This diagram depicts the differences in cellular and humoral immune responses in three different anatomical locations. The picture also shows the change/maturation over time highlighting a clear shift in immunological phenotypes. IM intramuscular; IN intranasal; PBMCs peripheral blood mononuclear cells.
